# Loss of SR-BI Down-Regulates MITF and Suppresses Extracellular Vesicle Release in Human Melanoma

**DOI:** 10.3390/ijms20051063

**Published:** 2019-03-01

**Authors:** Katharina Kinslechner, Birgit Schütz, Martina Pistek, Philipp Rapolter, Hans P. Weitzenböck, Harald Hundsberger, Wolfgang Mikulits, Johannes Grillari, Clemens Röhrl, Markus Hengstschläger, Herbert Stangl, Mario Mikula

**Affiliations:** 1 Center for Pathobiochemistry and Genetics, Medical University of Vienna, 1090 Vienna, Austria; katharina.kinslechner@meduniwien.ac.at (K.K.); birgit.schuetz@meduniwien.ac.at (B.S.); martina.pistek@gmx.net (M.P.); rapolter.philipp@gmx.at (P.R.); clemens.roehrl@meduniwien.ac.at (C.R.); markus.hengstschlaeger@meduniwien.ac.at (M.H.); herbert.stangl@meduniwien.ac.at (H.S.); 2 Medical and Pharmaceutical Biotechnology, IMC University of Applied Sciences, 3500 Krems, Austria; hans.weitzenboeck@fh-krems.ac.at (H.P.W.); harald.hundsberger@fh-krems.ac.at (H.H.); 3 Department of Medicine I, Division: Institute of Cancer Research, Comprehensive Cancer Center, Medical University of Vienna, 1090 Vienna, Austria; wolfgang.mikulits@meduniwien.ac.at; 4 Department of Biotechnology, BOKU -University of Natural Resources and Life Sciences, 1190 Vienna, Austria; johannes.grillari@boku.ac.at

**Keywords:** SCARB1, pigmentation, secretory pathway, melanoma metastasis, cMET, extracellular vesicles

## Abstract

Melanoma is a skin tumor with a high tendency for metastasis and thus is one of the deadliest cancers worldwide. Here, we investigated the expression of the scavenger receptor class B type 1 (SR-BI), a high-density lipoprotein (HDL) receptor, and tested for its role in melanoma pigmentation as well as extracellular vesicle release. We first analyzed the expression of *SR-BI* in patient samples and found a strong correlation with *MITF* expression as well as with the melanin synthesis pathway. Hence, we asked whether SR-BI could also play a role for the secretory pathway in metastatic melanoma cells. Interestingly, gain- and loss-of-function of SR-BI revealed regulation of the proto-oncogene MET. In line, SR-BI knockdown reduced expression of the small GTPase RABB22A, the ESCRT-II protein VPS25, and SNAP25, a member of the SNARE complex. Accordingly, reduced overall extracellular vesicle generation was detected upon loss of SR-BI. In summary, SR-BI expression in human melanoma enhances the formation and transport of extracellular vesicles, thereby contributing to the metastatic phenotype. Therapeutic targeting of SR-BI would not only interfere with cholesterol uptake, but also with the secretory pathway, therefore suppressing a key hallmark of the metastatic program.

## 1. Introduction

The scavenger receptor class B type 1 (SR-BI, gene name *SCARB1*) has emerged as a novel tumor marker, since its expression in different types of tumor cells has been associated with progression of the disease [1]. Studies showed reduced cell proliferation upon inhibition of SR-BI in human neuroblastoma, prostate carcinoma, clear-cell renal cell carcinoma and breast cancer cell lines [2,3,4,5]. The analysis of patient samples from prostate, breast and nasopharyngeal carcinoma highlighted a correlation between high SR-BI expression and worsening of the disease [6,7,8,9].

SR-BI is a so-called high-density lipoprotein (HDL) receptor [10,11,12] and known for its role in reverse cholesterol transport, namely the selective transfer of the cholesterol moiety to the hepatocytes. In contrast to the low-density lipoprotein receptor pathway, in which the endocytosed particle is completely dismantled, the HDL particles are not degraded, but transported to multivesicular bodies from which they can be exocytosed again [13,14].

Thus, the question arises as to why tumor cells express this specialized receptor and the related functional effects.

Among all tumors, melanoma represents a highly aggressive tumor entity and SR-BI plays an important role in maintaining the metastatic phenotype in this tumor [15]. Specifically, this includes the process of the epithelial-to-mesenchymal transition (EMT), migration, invasion and increased cellular glycosylation. Therefore, besides its role in mediating selective cholesteryl ester uptake from HDL particles to cells, SR-BI facilitates tumor promotion.

The secretory pathway is a key cellular process determining melanoma aggressiveness and has a major impact on clinical outcome. It involves membrane trafficking at the endoplasmic reticulum and Golgi as well as the sorting of proteins to the cytoplasm membrane. Here we investigated the involvement of SR-BI in the secretory phenotype of melanoma. By testing a large patient cohort, we identified the co-expression of SR-BI with the MITF transcription factor. During mammalian development MITF promotes the transition of precursor cells to melanoblasts [16]. In adult melanocytes, MITF is mainly known to regulate melanosome formation and melanin production [17,18]. MITF governs the melanocyte-specific melanin synthesis pathway with tyrosinase as an essential enzyme [19], and in melanoma it is one of the main drivers of proliferation and invasiveness [20]. Interestingly, melanoma cells display varying amounts of MITF, which can be used to identify different subpopulations within melanoma lesions [21,22,23]. A well-known MITF target is the proto-oncogene MET, which we found to be regulated by SR-BI knockdown as well as over-expression. MET receptor has been shown to correlate with the metastatic phenotype [24]. Additionally, MET governs exosome release in melanoma leading to enhanced metastatic colonization [25,26]. Accordingly, we investigated whether SR-BI is also involved in the generation of extracellular vesicles.

## 2. Results

### 2.1. SR-BI Expression Pattern in Melanoma Is Associated with the Pigmentation Pathway

**Figure 1:**
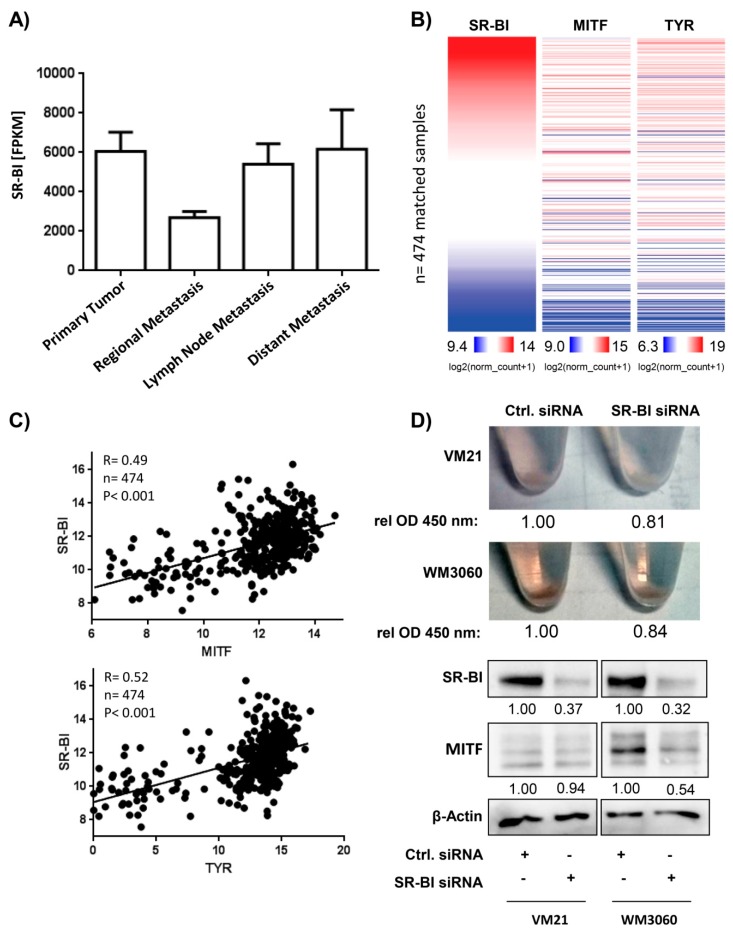
Scavenger receptor class B type 1 (SR-BI) is involved in the melanoma pigmentation pathway. (**A**) Expression levels of SR-BI in different tumor samples from melanoma patients. Mean with 95% confidence intervals is shown. FPKM = fragments per kilobase million. (**B**) Co-expression pattern of *SR-BI* with *MITF* *and* tyrosinase (*TYR*) from 474 matched melanoma samples. (**C**) Correlation of *SR-BI* with *MITF* (upper graph) and *TYR* (lower graph) expression in patient samples derived from metastatic lesions by the TCGA consortium. (**D**) Representative images of pelleted cells treated either with control siRNA or SR-BI siRNA. Immunoblotting of pigmented human melanoma cell lines VM21 and WM3060 after SR-BI knockdown showing SR-BI and MITF. β-ACTIN was used as loading control. rel OD = relative optical density.

When primary melanoma metastasizes, the most proximal sites are regional cutaneous metastases, followed by lymph node metastatic sites and finally distant metastatic sites. In order to display the distribution of *SR-BI* expression at each location, we used data derived from the Cancer Genome Atlas (TCGA) consortium and found highest *SR-BI* expression in primary lesions as well as in distant metastatic sites (Figure 1A). Interestingly, sorting for *SR-BI* expression intensity from highest to lowest revealed that *MITF* and its target tyrosinase (gene name *TYR*) segregated accordingly (Figure 1B). The correlation analysis confirmed this result (Figure 1C). To test whether SR-BI is necessary for the pigmentation phenotype in melanoma we used the melanoma cell line VM21, derived from early growth phase melanoma, and WM3060, derived from metastatic melanoma, to investigate the effect of *SR-BI* siRNA treatment. Control or SR-BI-depleted human melanoma cells were pelleted and displayed diminished brown pigmentation (Figure 1D, upper panel). Accordingly, the MITF protein was highly expressed in WM3060 cells and showed a strong reduction after SR-BI knockdown. VM21 cells, which expressed only moderate amounts of MITF, displayed only a slight reduction after knockdown of SR-BI (Figure 1D, lower panel).

### 2.2. SR-BI Controls the Proto-Oncogene and Vesicle-Release-Driver MET

**Figure 2:**
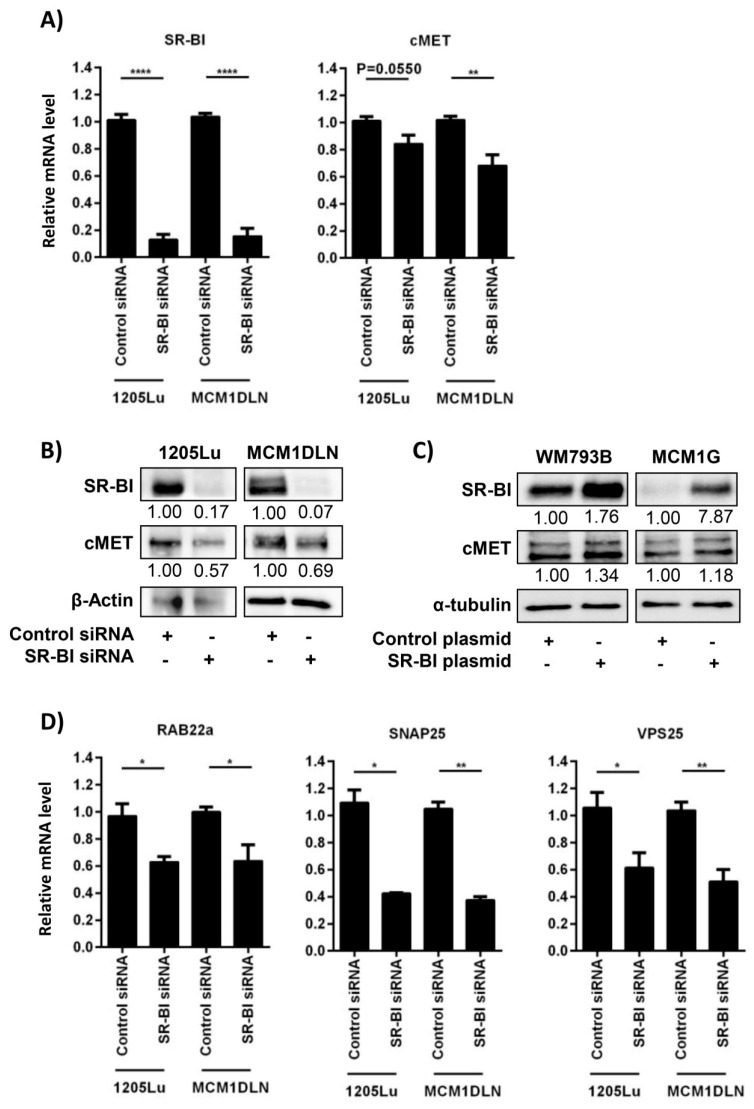
SR-BI depletion reduces the proto-oncogene and vesicle-release-driver cMET. (**A**) mRNA expression of *SR-BI* (1205Lu **** *P* < 0.0001, MCM1DLN **** *P* < 0.0001) and *MET* (1205Lu *P* = 0.0550, MCM1DLN **P = 0.0041) in two human metastatic melanoma cell lines. (**B**) Immunoblotting of SR-BI and MET after SR-BI knockdown of two human metastatic melanoma cell lines. β-ACTIN was used as loading control. (**C**) Immunoblotting of SR-BI and MET after SR-BI over-expression of two primary melanoma cell lines. α-TUBULIN was used as loading control. (**D**) Relative expression levels of *RAB22a* (1205Lu * *P* = 0.0131, MCM1DLN * *P* = 0.0267), *SNAP25* (1205Lu * *P* = 0.0196, MCM1DLN ** *P* = 0.0068) and *VPS25* (1205Lu * *P* = 0.0305, MCM1DLN ** *P* = 0.0028) in two human metastatic melanoma cell lines.

MITF is a driver of melanin synthesis and can activate the transcription of MET, an important receptor tyrosine kinase involved in tumor cell growth and release of extracellular vesicles. We used two well-characterized human melanoma cell lines derived from metastatic sites and performed SR-BI knockdown. mRNA harvested 48 hours after siRNA treatment showed a reduction of *SR-BI* and down-regulation of the *MET* receptor (Figure 2A). Protein analysis showed a strong reduction of SR-BI and a concomitant MET reduction in both cell lines (Figure 2B). Over-expression experiments for SR-BI indicated that SR-BI is able to positively regulate MET expression (Figure 2C). Since the release of extracellular vesicles contributes to the malignant phenotype in metastatic melanoma, we next quantified the mRNA levels of regulatory proteins in the secretory pathway. SR-BI removal led to the diminished expression of *RAB22a*, *SNAP25* and *VPS25*, which suggested reduced generation and secretion of tumor-derived extracellular vesicles (Figure 2D).

### 2.3. Extracellular Vesicle Secretion Is Altered after SR-BI Inhibition

**Figure 3:**
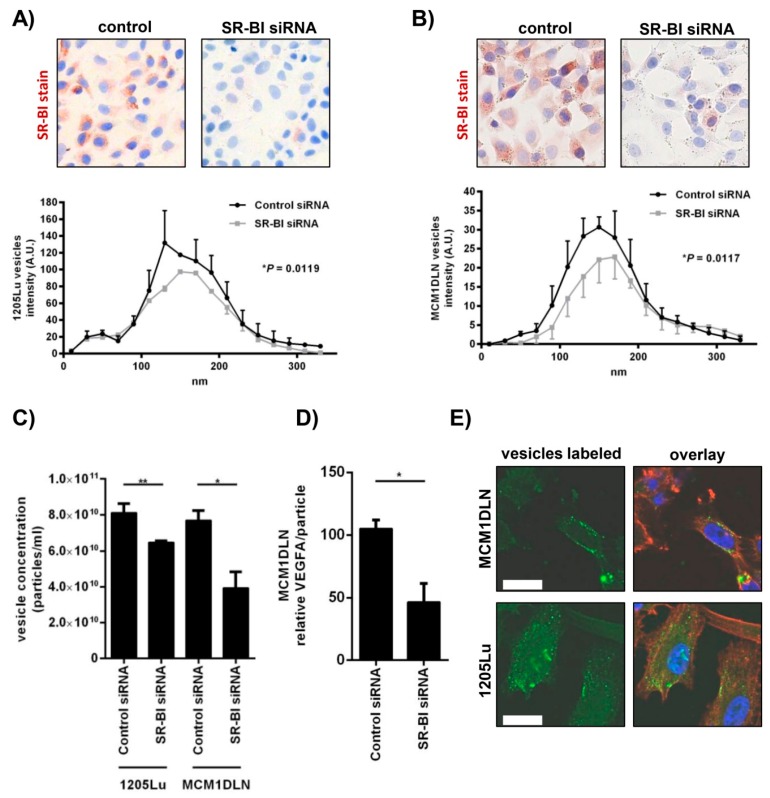
Extracellular vesicle secretion is inhibited after SR-BI knockdown. (**A** and **B**) 1205Lu and MCM1DLN cells were treated with SR-BI siRNA and the knockdown efficiency is shown on the protein level by immunocytochemistry (**upper panel**). The lower panel shows the extracellular vesicle distribution, isolated from the culture media, measured by Nanosight. (**C**) Extracellular vesicle concentration of two human metastatic melanoma cell lines (1205Lu ** *P* = 0.0087; MCM1DLN * *P* = 0.0232) after SR-BI knockdown was measured. (**D**) Relative VEGFA amount per vesicle of MCM1DLN cells after control siRNA or SR-BI siRNA treatment (* *P* = 0.0377). (**E**) Vesicles derived from MCM1DLN and 1205Lu were CFSE labeled and incubated on respective melanoma cell lines for 20 min. The cells were fixed and the labeled vesicles were visualized by confocal microscopy. Bar represents 10 µm. CFSE = green, phalloidin = red, DAPI = blue.

We isolated extracellular vesicles from the supernatant of control cells and SR-BI depleted cells of two metastatic human melanoma cell lines. In Figure 3A,B (upper panel) we show the knockdown efficiency as demonstrated by immunocytochemical staining for SR-BI. In Figure 3A,B (lower panel), we calculated the vesicle distribution as quantified by Nanosight of the isolated vesicles in both melanoma cell lines. The analysis revealed that the vesicle count per volume decreased by 20% and 50%, according to the cell model used (Figure 3C). Since vesicles are known to be the carriers of tumor promoting factors, we ruptured harvested vesicles by repeated freeze–thaw cycles and measured VEGFA by ELISA. Equal amounts of vesicles derived from control treated or SR-BI siRNA treated cells were used and a significant reduction of VEGFA was detected (Figure 3D). Finally, to test whether vesicles could attach to cells we labeled isolated vesicles with a fluorescent dye. Melanoma cells were incubated with labeled vesicles and 30 minutes after exposure the cells were formaldehyde fixed. Confocal image acquisition showed that green fluorescent vesicles of a very small size could be detected at the cellular membrane and within the cellular cytoplasm (Figure 3E).

### 2.4. SR-BI and MET Expression Correlates in Patient Data Sets

**Figure 4:**
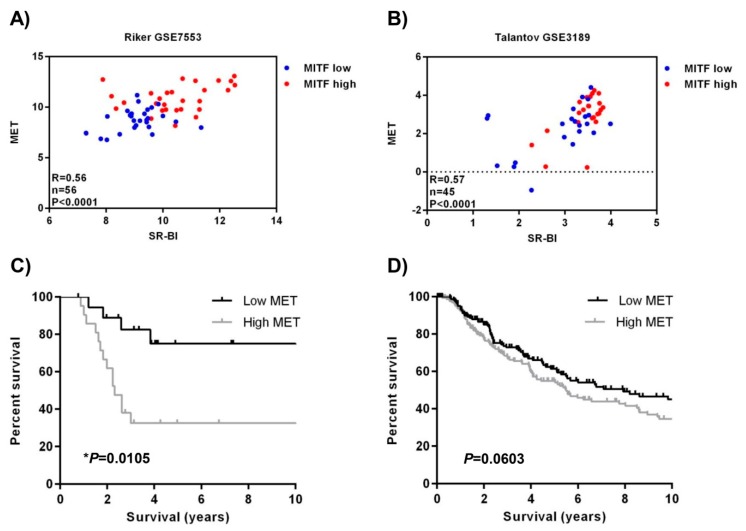
SR-BI and MET correlate in patients. (**A** and **B**) Patient sample analysis for correlation of *SR-BI* with *MET* and additional indication of *MITF*^high^ expression in red color and *MITF*^low^ expression in blue color. (**A**) Patient data set GSE7553. (**B**) Patient data set GSE3189. Data sets consisting only of melanoma biopsies, skin tissue and other cancer types were excluded in this analysis. (**C** and **D**) Ten-year follow-up Kaplan–Meier survival curves of the two data sets (**C**) GSE19234 (*n* = 44) and (**D**) SKCM-TCGA (*n* = 335). Grey line indicates high *MET* levels, black line indicates low *MET* levels. Values were calculated using a log-rank test.

To strengthen our finding of SR-BI regulating MITF as well as MET, we made use of two published melanoma whole genome expression studies. There we found a positive correlation between *SR-BI* and *MET* (Figure 4A,B). Additionally, we divided the data according to the median expression of *MITF* into low and high expression groups, indicated by blue and red dots, respectively. The data demonstrate that only the samples that expressed high amounts of *SR-BI* and *MET* also displayed high *MITF*. This is of particular interest since *MET* is a discriminator of patient survival, as can be seen in two mRNA data sets GSE19234 (Figure 4C) and SKCM-TCGA (Figure 4D) by Kaplan–Meier analysis. Our data indicate that high mRNA levels of *MET* led to a worsened survival outcome for patients, associated with high *MITF* and *SR-BI* expression.

## 3. Discussion

We have shown that SR-BI in human melanoma is important for the pigmentation phenotype driven by the MITF transcription factor. Furthermore, SR-BI regulated the expression of the MET proto-oncogene, and the loss of SR-BI resulted in the down-regulation of molecules involved in the secretory pathway. Measurements of extracellular vesicle size and density revealed that without SR-BI, vesicle size increased and vesicle generation per donor cell was reduced. Importantly, the released vesicles contained less of the pro-tumorigenic factor VEGFA when derived from SR-BI knockdown cells. Hence, we could demonstrate that SR-BI plays an important role in metastatic melanoma by enhancing the secretory process encompassing extracellular vesicles as well as tumor-derived growth factors.

Melanin synthesis driven by MITF depends on the mammalian secretory pathway and is highly sensitive to defects in the glycosylation pathway as well as defects in ER, Golgi and Post-Golgi transport [27]. We have shown that the loss of SR-BI reduces the ability to glycosylate proteins at the ER-Golgi, which resulted in the reduced secretion of VEGFA [15]. Hence our data presented here further link SR-BI to the secretory pathway, which includes the pigmentation process, but which also includes pro-tumorigenic extracellular vesicle formation and release.

Interestingly, MITF represents the major regulator of the pigmentation pathway, and at the same time amplification of the *MITF* locus was shown to be associated with melanoma metastasis [28]. Therefore, MITF harbors a wide range of functions including melanocytic lineage restriction, cell cycle regulation, survival and migration. Since we were able to show that MITF expression depends on the presence of SR-BI, it is interesting to note that SR-BI could be considered an upstream regulator of MITF. This regulation might occur via the AKT signaling pathway, which has been shown to be induced upon cholesterol transport by SR-BI in different cellular models [29,30,31]. Studies in melanocytes have shown that AKT activation is necessary for GSK3beta inhibition leading to MITF expression [32]. Additionally, the application of excess cholesterol, which can be transported by SR-BI [33], induced pigmentation and upregulation of MITF in human melanocytes and melanoma cells [34]. High MITF activity leads to expression of its target genes. Among these, SR-BI has been identified [35]. Taken together, this implies that SR-BI represents a potent inducer of MITF and that MITF itself is connected to SR-BI by a positive feedback loop, which could explain the strong correlation we observed in the patient samples.

Another important downstream target of MITF is the MET receptor [36]. MET expression together with dopachrome tautomerase (TYRP2), which is part of the melanin synthesis process, is part of a melanoma signature and prognostic for tumor progression [25]. Thus, the pigmentation process is linked to vesicle generation and release, which is crucially involved in tumor progression.

The budding of the plasma membrane during ectocytosis requires Rab GTPases, the endosomal sorting complex (ESCRT) and possibly also SNARE proteins. The HDL components phosphatidyl-inositols and phosphatidyl-ethanolamine also play an important role in extracellular vesicle budding [37]. We detected significantly fewer extracellular vesicles, characterized by a diameter bigger than 90 nm, upon the depletion of SR-BI. In line, we were able to identify the down-regulation of *RAB22A*, *VPS25* and *SNAP25*. RAB22A is important for early endosomal sorting and its over-expression promotes melanoma growth [38]. VPS25 is a member of the ESCRT-II complex and is able to recruit ESCRT-III, which executes vesicle budding and scission [39]. SNAP25 is involved in the regulation of neurotransmitter release and is localized in the presynaptic plasma membrane. Since melanoma cells show a neuronal phenotype it could be concluded that SNAP25 is important for neuronal-like vesicle generation. We suggest that extracellular vesicles released by melanoma cells could lead to the autocrine stimulation of tumor cells or even be involved in preparing a metastatic niche by fusing with proximal or distal stromal cells.

Taken together, SR-BI influences vesicle generation and release at multiple levels. This includes the role of SR-BI in supplying cancer cells with cholesterol as well as its role in ER-Golgi-associated protein glycosylation. Here we identified MITF, the MET receptor as well as several members of the secretory pathway as being regulated by SR-BI in human melanoma cells. Moreover, patient sample analysis confirmed the correlation of SR-BI with MITF and MET in metastatic melanoma lesions. Our data pinpoint SR-BI as an important functional node for enabling not only melanin synthesis and potential melanosome formation, but also for generating tumor promoting extracellular vesicles. Therefore, our results show that SR-BI represents an important player in disease progression in melanoma. We suggest that the inhibition of SR-BI will interfere with the metastatic phenotype and in combination with state of the art treatment might lead to regression of the disease.

## 4. Materials and Methods 

### 4.1. Cell Culture and Melanin Measurement

Patient-derived melanoma cell model MCM1 has been characterized previously [24]. Human melanoma cell lines 1205Lu was purchased from ATCC and WM3060 from Coriell. The cell line VM21 was a gift from Walter Berger and has been described previously [40]. All cells were cultivated under standard conditions and maintained in melanoma isolation medium (MIM) supplemented with 2% FCS, except the cell line VM21, which was grown in RPMI (Lonza, Basel, Switzerland) containing 10% FCS. MIM contains 80% MCDB153, 20% Leibovitz′s L-15, 1.23% sodium bicarbonate solution, 0.5 ng/mL epidermal growth factor, 5 μg/mL human insulin, 1.68 mM calcium chloride (all from Sigma-Aldrich, St. Louis, MO, USA), 50 mg/l streptomycin sulfate, 30 mg/L penicillin (both from GE Healthcare, Buckinghamshire, United Kingdom), and 2 μg/mL ciprofloxacin (Sigma). Pelleted cells were imaged by a color camera. The pixel density of the identical pellet area was calculated in the blue color channel at 450 nm.

### 4.2. Transient SR-BI Overexpression

Human melanoma cells were seeded in a 6-well plate and transduced with PEI (Polysciences Inc., Warrington, PA, USA) and 3 µg wildtype plasmid or plasmid containing human SR-BI (pcDNA™5/FRT Vector, Invitrogen, Paisley, UK). Overexpression efficiency was tested by Western blot analysis.

### 4.3. siRNA Knockdown

Reagents for siRNA knockdown were obtained from Dharmacon (GE Healthcare). Cells were transfected with DharmaFECT 1 and 5 nM ON-TARGETplus SMARTpool siRNA against *SCARB1* (L-010592) or non-targeting siRNA (D-001810) for 24 hours followed by mRNA isolation (72 h post-transfection) and protein isolation (96 h post-transfection). Knockdown efficiency was tested by RT-PCR and Western blot analysis.

### 4.4. RNA Isolation and Real-Time PCR

RNA was isolated according to the manufacturer’s instructions (peqGOLD total RNA Kit). cDNA synthesis and polymerase chain reaction (PCR) were performed. Primer pairs are listed in Table 1. Values were normalized to human *ACTB* mRNA expression. Results were quantified using the Delta C(T) method.

### 4.5. Western Blotting

Cells were lysed with RIPA buffer (Cell Signaling, Frankfurt am Main, Germany) on ice, and SDS–PAGE and Western blotting was performed according to standard protocols. Nitrocellulose membranes (Biorad, Munich, Germany) were incubated with the antibodies listed in Table 2. Semi-quantitative densitometric analyses of the detected bands were performed with the corresponding software from Biorad according to standard procedures.

### 4.6. Immunocytochemistry and Immunofluorescence

Cells grown in chamber slides were fixed with 4% formaldehyde, washed with PBS, treated for 5 min with 1% Triton X-100, washed, blocked with 1% bovine serum albumin and then stained with specific antibodies at 4 °C overnight (see Table 2). IF images were taken with a Leica TCS SP8 microscope (Leica Microsystems, Wetzlar, Germany).

### 4.7. ELISA

Vascular endothelial growth factor (VEGF) was measured from supernatant using the Human VEGF Mini Development Kit from PeproTech (Vienna, Austria) as described by the manufacturer. 2,2′-azino-bis(3-ethylbenzothiazoline-6-sulphonic acid) was used as substrate (Sigma-Aldrich).

### 4.8. Vesicle Isolation 

The 60 × 10^6^ human melanoma cells were grown in serum-free MIM for 48 h. Collected supernatant fractions were filtered with a 0.2 µm filter (Sarstedt, Nümbrecht, Germany). Vesicles were pelleted by ultracentrifugation at 34800 rpm for 70 min at 4 °C (Beckman Coulter 55.2 Ti rotor, Krefeld, Germany). The pellet, containing vesicles, was resuspended in 100 µl PBS. Vesicle concentration and size were measured using the Nanosight technology equipped with a blue laser (405 nm) for real-time characterization of the vesicles.

### 4.9. Statistical Analysis

All values are given as means ± standard error of the mean (S.E.M.), except when noted otherwise. Figures were analyzed and drafted with GraphPad Prism^®^ 6. Western blots and RT-PCRs were repeated at least two times with at least two replicates. Kaplan–Meier plots and scatter plots were generated from the publicly available data sets GSE19234 [41], GSE7553 [42], GSE3189 [43] and the SKCM-TCGA study on cutaneous melanoma [44]. Pearson correlation analysis was performed to calculate the P-values in displayed scatter blots. Differences in gene expression were assessed for statistical significance by an unpaired two-tailed t test. Log-rank (Mantel–Cox) test was performed for Kaplan–Meier analysis. *P* < 0.05 was defined as statistically significant and *P*-values are displayed as follows: * *P* < 0.05; ** *P* < 0.01; *** *P* < 0.001; and **** *P* < 0.0001.

## Figures and Tables

**Table 1:** Table of primers.

Primer Name	5′ -> 3′ Forward	5′ -> 3′ Reverse
β-Actin (ACTB)	CTATCCAGGCTGTGCTATCCCTGT	CCTTAATGTCACGCACGATTTCC
MET	GGCGACAGCTGACTTGCTGAGAG	GTAAACAGGAGCACGAGGATGCCAG
RAB22A	GCTTCGACAGCATGGCCCAC	AGATGGCAGGTTGGCGTCAGT
SNAP25	CTGCGGGCTTTGTGTGTGTCC	TGACGGAGGTTCCCGATGATGC
SR-BI (SCARB1)	GTACGTCCTCCTGGCGCTGG	GCAGCACAGAGCCCTTGGGA
VPS25	CATGTGGCGGAGGCCAGAAGA	GCCCGCAGTAGAGTGGCTTCA

**Table 2:** Table of antibodies.

Antibody	Company	Cat. No.	ICC/IF ^1^	WB ^2^
α-tubulin	Calbiochem	CP06		1:5000
β-Actin	Santa Cruz	sc-47778		1:1500
MET	Cell Signaling	#8198	1:200	1:1000
MITF	Abcam	ab12039		1:1000
SR-BI	BDBiosciences	610882	1:500	1:2000
Biotinylated anti-rabbit	Vector Laboratories	BA-1100	1:500	
Biotinylated anti-mouse	Vector Laboratories	BA-2000	1:500	
Peroxidase conj. anti-rabbit	Thermo Scientific	31460		1:5000
Peroxidase conj. anti-mouse	Thermo Scientific	31430		1:5000
Dylight488 anti-rabbit	Abcam	ab96923	1:400	
Dylight550 anti-mouse	Abcam	ab96876	1:400	

^1^ Immunocytochemistry (ICC) and immunofluorescence (IF), ^2^ Western blotting (WB).

## Abbreviations

ATCC	American Type Culture Collection
CFSE	Carboxyfluorescein succinimidyl ester
DAPI	4′,6-diamidino-2-phenylindole
FCS	fetal calf serum
FPKM	Fragments Per Kilobase Million
ICC	immunocytochemistry
IF	immunofluorescence
MIM	melanoma isolation medium
rel OD	relative optical density
RT-PCR	real-time polymerase chain reaction
SR-BI	scavenger receptor class B type I
TCGA	The Cancer Genome Atlas
